# Bilateral thoracoscopic sympathectomy for primary hyperhydrosis: a review of 335 cases

**DOI:** 10.5830/CVJA-2013-007

**Published:** 2013-06

**Authors:** Murat Öncel, Güven Sadi Sunam, Esref Erdem, Yüksel Dereli, Bekir Tezcan, Kazim Gürol Akyol

**Affiliations:** Department of Thoracic Surgery, Selcuklu Medicine Faculty, Selcuk University, Konya, Turkey; Department of Thoracic Surgery, Selcuklu Medicine Faculty, Selcuk University, Konya, Turkey; Department of Thoracic Surgery, Selcuklu Medicine Faculty, Selcuk University, Konya, Turkey; Department of Cardiovascular Surgery, Numune State Hospital, Konya, Turkey; Department of Thoracic Surgery, Numune State Hospital, Konya, Turkey; Department of Thoracic Surgery, Numune State Hospital, Konya, Turkey

**Keywords:** primary hyperhydrosis, bilateral thoracoscopic sympathectomy, VATS

## Abstract

**Objective:**

The goal of this retrospective study was to evaluate the outcomes of bilateral video-assisted thoracoscopic sympathectomy for primary hyperhydrosis.

**Methods:**

Between January 2007 and December 2011, a total of 335 patients (192 male, 143 female, mean age 28.3 years) who underwent bilateral thoracoscopic sympathectomy for primary hyperhydrosis were reviewed retrospectively.

**Results:**

Hyperhydrosis occurred in the palmar and axillary region in 175 (52.23%) patients, in only the palmar region in 52 (15.52%), in the craniofacial region in 44 (13.13%), in only the axillary region in 42 (12.53%), and in the palmar and pedal regions in 22 (6.56%) patients. Bilateral thoracoscopic sympathectomy was performed in all patients. The mean follow-up period was 24 (6–48) months. The initial cure rate was 95% and the initial satisfaction rate was 93%. There was no mortality in this study. The complication rate was 15.82% in 53 patients.

**Conclusion:**

Video-assisted thoracoscopic sympathectomy for the treatment of primary hyperhydrosis was effective, with low rates of morbidity and mortality. Despite the appearance of postoperative complications, such as compensatory sweating, patient satisfaction with the procedure was high and their quality of life improved.

## Abstract

Primary hyperhydrosis is a disorder characterised by excessive diffuse or localised sweating. Although its pathophysiology is unknown, it is believed to be caused by hyper-stimulation or over-activity of the sympathetic nervous system.^1^ It most often affects the palms of the hands, the axillae, or the face. Although not life-threatening, hyperhydrosis causes educational and occupational difficulties, as well as psychological and social problems. Medical treatment is often ineffective and the response is usually transient.

Thoracoscopic sympathectomy is the current standard treatment for primary hyperhydrosis. This procedure is easy, safe, fast, effective and minimally invasive. The most common side effect of this method is compensatory sweating (CS) and it is believed to be due to a thermoregulatory mechanism.^2^ In this study, we present our experience with this technique.

## Methods

A total of 335 patients underwent one-stage bilateral thoracoscopic sympathectomy for treatment of primary palmar hyperhidrosis, by the same surgical team. All patients had failed to respond to adequate medical treatment and were referred for surgery.

Selection criteria included absence of previous thoracic surgery, severe and debilitating primary palmar hyperhydrosis, repercussions on social life, repercussions on professional activity, effect on intimate relationship, inefficacy of medical treatment, and patient motivation and determination. Each patient signed an informed consent after having been carefully educated on the possible side effects, such as transient compensatory dry-hand sweating, intercostal pain, Horner syndrome, pneumothoax and haemopneumothorax.

General anaesthesia using the single lung-isolation technique with a double-lumen endotracheal tube was used in all patients. The patients were positioned in a semi-sitting position with their arms in abduction. Patients were placed in a semi-prone position for each side of the procedure, with the ipsilateral arm abducted and a mild anti-Trendeleburg position.

Monitoring included saturation, blood pressure and electrocardiogram. In those patients undergoing general anesthesia, one-lung ventilation was administered using a double-lumen endotracheal tube. Thoracoscopic ports were placed, respectively, in the fourth intercostal space on the mid-axillary line and the third intercostal space anterior to the mid-axillary line.

All patients underwent division of their sympathetic chain using controlled intermittent electrocautery after identifying the first rib. The T1 chain was kept for Horner syndrome surgical ablation of the sympathetic chain between T2 and T3, and electrocautery ablation of the accessory branches and Kuntz nerve, when present, was performed in all cases to prevent relapse of the health status immediately before and six months after surgery. In five patients, very thin adhesions were encountered that could easily be eliminated by electrocautery.

A postoperative chest X-ray was performed in the recovery room to verify the absence of a significant haemothorax or pneumothorax. A temporary intra-operative paediatric chest tube was inserted into the chest during closure of the incisional soft tissues but was removed before tying down the skin closure suture.

## Results

Video-assisted thoracoscopic sympathectomies (670) were performed in 335 patients with severe primary hyperhydrosis (Table 1). Of the 113 patients, 57.31% (*n* = 192) were female, 42.69% (*n* = 143) were male, and the mean age was 28.32 (range 18–54) years. Palmar and axillary hyperhydrosis were the most common symptoms. Hyperhydrosis was observed in the palmar and axillary region in 175 (52. 23%) patients, only in the palmar region in 52 (15.52%), in the craniofacial region in 44 (13.13%), only in the axillary region in 42 (12.53%), and in the palmar and pedal regions in 22 (6.56%) patients.

**Table 1:** Patients Characteristics

Mean age	28.32 (range 18–54) years	
Gender	Male	42.69% (*n* = 143)
	Female	57.31% (*n* = 192)
Localisation of hyperhydrosis	Palmar and axillary (T3–T4 resected)	52.23% (*n* = 175)
	Isolated palmar (T3 resected)	15.52% (*n* = 52)
	Craniofacial (T2 resected)	13.13% (*n* = 44)
	Isolated axillary (T3 resected)	12.53% (*n* = 42)
	Palmar and pedal (T3–T5 resected)	6.56% (*n* = 22)
Complications	Compensatory sweating	10.14% (*n* = 34)
	Hyperesthesia at the incision area	2.08% (*n* = 7)
	Pneumothorax	1.49% (*n* = 5)
	Bleeding	0.89% (*n* = 3)
	Horner’s syndrome	0.59% (*n* = 2)
	Emphysema	0.29% (*n* = 1)
	Chylothorax	0.29% (*n* = 1)
Re-operation	0.89% (*n* = 3)	
Recurrence	0	
Mortality	0	

Bilateral video-assisted thoracoscopic sympathectomy was performed in all patients. T2 resection was performed in 44 patients with craniofacial hyperhydrosis. T3 resection was performed in 94 patients with only palmar and only axillary hyperhydrosis. T3–T4 resection was performed in 175 patients with palmar and axillary hyperhydrosis. T3–T5 resection was performed in 22 patients with palmar and pedal hyperhydrosis. The mean operating time was 51 (range 37–78) minutes. Re-operation required for 3 (0.89%) patients due to bleeding.

Usually, patients were discharged within 24–48 hours after operation. The mean follow-up period was 24 (range 6–48) months. Most of the patients presented with an improvement in primary hyperhydrosis. The initial cure rate was 95% and the initial satisfaction rate was 93%. There was no mortality in this study. No recurrence was reported at the follow-up period. The complication rate was 15.82% in 53 patients. Compensatory sweating was the most frequent complication in 34 (10.14%) patients.

## Discussion

Primary hyperhydrosis is a disorder characterised by excessive sweating. Somewhat more frequent in women, there is an obvious family predisposition and its incidence is higher in certain populations (Asians and Sephardic Jews), representing 1% of the population.^3^ The condition is bilateral, symmetrical and is sometimes related to or exacerbated by emotional or seasonal situations. Although there are generalised forms, focal hyperhydrosis is the most frequent presentation. Palmar and axillary hyperhydrosis are the most common, followed by plantar hyperhydrosis.

Although its pathophysiology is unclear, it is believed to be caused by hyper-stimulation or over-activity of the sympathetic nervous system that passes through the upper thoracic ganglia.^1^ The diagnosis of primary focal hyperhydrosis is based on symptoms, and supported by a specific clinical history. Help is needed to distinguish focal from generalised hyperhydrosis. The Multi-Specialty Working Group on Hyperhydrosis in the United States has proposed some criteria for the diagnosis of focal hyperhydrosis (Table 2).^4^

**Table 2:** Criteria For Primary Focal Hyperhydrosis Diagnosis

A	Focal, visible, excessive sweating for a period of at least six months with no known secondary cause
B	At least one of the following characteristics:
	• bilateral and symmetrical
	• frequency of at least one episode per week
	• interferes with daily activities
	• presentation before the age of 25
	• family history
	• cessation of excessive sweating during sleep.

Although not life-threatening, hyperhydrosis causes educational and occupational difficulties, as well as psychological and social problems. Medical treatment is often unsuccessful and the response is usually transient. Therapeutic options for its management include topical anti-perspirants, anti-cholinergic drugs, iontophoresis and recently, botulinum toxin injections.^5^,^6^

Interruption of the sympathetic innervation of the ecrine sweat glands via the upper thoracic ganglia during surgery is the best procedure for hyperhydrosis. Surgery of the thoracic sympathetic nervous system has been performed since the beginning of the 20th century.^7^ Thoracoscopic sympathectomy was first described in 1942 by Hughes, and remained rare until the introduction of video-endoscopic techniques in the 1980s.^8^ Since then it has become the preferred method of treatment of primary hyperhydrosis of the palms, axillae, and more recently for facial blushing. Its popularity has grown so much that the technique is now used around the world.

Bilateral endoscopic sympathectomy can be performed by different surgical and anaesthesiological techniques. Giving the patient the chance to achieve both functional and aesthetic results with minimal risk and discomfort, together with an excellent postoperative quality of life is the gold standard.^9^ Different techniques are used for intra-operative ventilation of these patients. Orotracheal intubation with a double-lumen endotracheal tube is most commonly used but there are reports of the use of ventilation with a laryngeal mask, as well as surgery under sedation with spontaneous ventilation, with satisfactory results.^10^

In recent years, numerous articles advocating diverse surgical techniques (ablation, resection, interruption by clips, etc) for accessing the thoracic sympathetic chain have been published. Hashmonai *et al.* compared surgical techniques for the treatment of hyperhydrosis (resection or electrocoagulation) based on a review of the studies published between 1974 and 1999.^11^ Although resection seems to provide better results, the authors noted that the majority of surgeons tend to use electrocoagulation, perhaps because of its simplicity and shorter surgical time.

Thoracoscopic resection of the upper thoracic sympathetic chain has been defended by numerous authors over the last decade as the treatment of choice for hyperhydrosis.^12^,^13^ As previously reported by Garcia and Espania in all cases via video-assisted thorascopic surgery: one or more ganglia between T2 and T5 are usually resected depending on the area affected by hyperhydrosis.^14^ They recommend intervention on the T2 ganglia for craniofacial hyperhydrosis or rubor, on the T3 ganglia for palmar hyperhidrosis, and on the T3 and T4 ganglia for combined palmar and axillary hyperhydrosis.

Using the Lin-Teleranta classification as a basis, the results of various authors can be analysed, finding enough differences between them to not be able to categorically indicate the precise levels for action, but at the same time finding enough evidence to recommend specific cut levels:

• Patients with facial flushing and/or sweating, cut level T2 and/or T3.
• Patients with palmar hyperhydrosis, cut level T3 and/or T4.
• Patients with axillary hyperhydrosis, cut level T4 or T4–T5.^15^-^17^

In our study, T2 resection was performed in 44 patients with craniofacial hyperhydrosis, T3 resection was performed in 94 patients with only palmar and only axillary hyperhydrosis, T3–T4 resection was performed in 175 patients with palmar and axillary hyperhydrosis, and T3–T5 resection was performed in 22 patients with palmar and pedal hyperhydrosis.

Studies of patient series in which primary hyperhydrosis has been treated surgically reveal a high degree of patient satisfaction. The best results are reported by Lardinois and Ris, with improvement in quality of life in 94.6% of the patients, and Cohen *et al.*, with a satisfaction rate of 98.2%.^18^,^19^ The most frequent postoperative complication is the appearance of pneumothorax, which is usually small and requires pleural drainage in 30% of cases. In a few patients, pleural effusion, haemothorax or chylothorax can occur.^20^

In general, pain is moderate and is resolved with conventional analgesics over a maximum period of two to four weeks. Horner syndrome appears permanently in less than 0.5% of cases. Other less frequent complications are subcutaneous emphysema, infection of the surgical wound, the presence of segmental atelectasis and transitory lesions of the brachial plexus.^21^

In our study, most of the patients presented with an improvement in primary hyperhydrosis. The initial cure rate was 95% and the initial satisfaction rate was 93%. There was no mortality and no recurrence was reported at the follow-up period. The complication rate was 15.82% in 53 patients. CS was the most frequent complication in 34 (10.14%) cases.

CS remains the most common and most disabling complication of video-assisted thoracoscopic sympathectomy and it is believed to be due to a thermoregulatory mechanism.^2^ CS is defined as intense sweating in other anatomical areas after sympathectomy. The most frequent sites are the thorax, back, abdomen and inguinal region.^22^ This situation can change over time and is usually difficult to evaluate. The reported frequencies vary considerably, with conflicting views as to its severity and predisposition. Factors such as geographic location, working environment, climatic conditions (temperature and humidity), together with heterogeneity of the population can also affect the incidence of CS.

According to the series reviewed by Dumont, the mild form oscillates between 15 and 90% and the severe form between 1 and 30%.^21^ According to some authors, the higher up the sympathectomy is done (T2) and the more extensive the resection (T2–T5), the greater the probability of there being serious CS.^20^,^21^ We had 34 cases with compensatory sweating. In our experience, if you protect the T2 ganglion, you have less reflex compensatory sweating.

## Conclusion

Bilateral video-assisted thoracic sympathectomy is currently a standard surgical technique to treat primary hyperhydrosis because it is a safe, easy, fast, effective and minimally invasive method. Despite the appearance of postoperative changes such as CS, patient satisfaction with the procedure is high and their quality of life improves.
